# Synergistic interactions of repurposed drugs that inhibit Nsp1, a major virulence factor for COVID-19

**DOI:** 10.1038/s41598-022-14194-x

**Published:** 2022-06-17

**Authors:** Hung-Teh Kao, Andrew Orry, Michael G. Palfreyman, Barbara Porton

**Affiliations:** 1 Sypherion LLC, Providence, RI 02903 USA; 2 Molsoft LLC, San Diego, CA 92121 USA; 3 Palfreyman BioPharm Advisors, LLC, St. Petersburg, FL 33701 USA

**Keywords:** Biotechnology, Drug discovery, Diseases, Medical research, Molecular medicine

## Abstract

Nsp1 is one of the first proteins expressed from the SARS-CoV-2 genome and is a major virulence factor for COVID-19. A rapid multiplexed assay for detecting the action of Nsp1 was developed in cultured lung cells. The assay is based on the acute cytopathic effects induced by Nsp1. Virtual screening was used to stratify compounds that interact with two functional Nsp1 sites: the RNA-binding groove and C-terminal helix-loop-helix region. Experimental screening focused on compounds that could be readily repurposed to treat COVID-19. Multiple synergistic combinations of compounds that significantly inhibited Nsp1 action were identified. Among the most promising combinations are Ponatinib, Rilpivirine, and Montelukast, which together, reversed the toxic effects of Nsp1 to the same extent as null mutations in the Nsp1 gene.

## Introduction

There is an urgent need for antiviral agents to treat COVID-19, which has claimed more than 6.2 million lives and over 510 million infections worldwide as of May 2, 2022, according to the World Health Organization (WHO) (https://covid19.who.int). Although treatments and vaccines have been developed to promote immune protection against infection, there are SARS-CoV-2 variants that evade current monoclonal antibody treatments^1–3^. These variants can partially escape protection induced by mRNA vaccines^3–5^. We also face waning immunity from vaccines^6^. Thus, there is a clear need for therapeutic interventions that act outside of the immune system to curb the threat of COVID-19.

Here, we report the identification of compounds that inhibit the action of Nsp1 (nonstructural protein 1). Nsp1 is one of the first proteins expressed from the SARS-CoV-2 genome upon infection of human cells and is a major pathogenicity factor^7–9^. Nsp1 is fundamentally an RNA binding protein that targets the 40S ribosomal subunit and the first stem-loop of the 5′UTR of viral RNAs^8,9^. Nsp1 also possesses significant protein–protein interactions that recruit translation factors and inhibit the export of mRNA from the nucleus^10^. Collectively, the outcome of these actions is the global inhibition of host cell mRNA translation while viral mRNA translation remains intact^7–13^. Accordingly, cellular innate defenses such as the induction of interferon is inhibited, while viral replication and assembly proceeds unhindered.

In addition to the key role played by Nsp1 during the initial stages of the viral life cycle, Nsp1 is the only protein expressed from the SARS-CoV-2 genome that leads to significant cell death^7^. The mechanism of cell death is apoptosis and occurs over the course of days after the expression of Nsp1^7^. The inhibition of cell protein synthesis likely contributes to apoptosis as well as the severe respiratory symptoms associated with COVID-19. Thus, therapeutics targeting Nsp1 could mitigate the symptoms of severe COVID-19 as well as slow the progression of the viral life cycle.

Another advantage to targeting Nsp1 is that the sequence of this protein has remained unaltered in all current SARS-CoV-2 variants of concern, including the alpha, beta, delta, epsilon, mu, and omicron variants. Therapeutics targeting Nsp1 would therefore target all such variants.

To rapidly identify compounds that act as inhibitors of Nsp1, we developed a cell-based cytopathic assay that takes advantage of Nsp1’s ability to induce cell death. The theoretical binding affinity of compounds targeting active regions of Nsp1 were stratified using in silico screening. Drugs that can be repurposed were prioritized for further investigation. No single drug in this group possessed the ability to completely reverse Nsp1 action. However, we identified combinations of drugs that act synergistically and were capable of significantly reducing Nsp1-mediated cell death. Moreover, the effective concentration of these drugs resides in the nanomolar range, which is clinically attainable in humans. Since the drugs have been previously used in humans, much is known about their side effects. Therefore, the testing of these drug combinations in clinical trials offers the possibility of alternative effective treatments for COVID-19.

## Results

### A rapid assay for Nsp1 action and validation with mutants of Nsp1

Nsp1 is the only viral gene product that significantly promotes apoptosis in lung cells during COVID-19 infection^7^. We exploited this property to design a cytopathic assay to quantitate the deleterious effects of Nsp1.

A synthetic gene encoding Nsp1 was constructed using sequences obtained from the original SARS-CoV-2 strain^14^. Capped mRNA was transcribed from the Nsp1 synthetic gene for expression in cultured cells. mRNA transfection was used to transiently express Nsp1 in cultured adherent cells, in order to simulate the conditions of viral infection and to ensure rapid expression of Nsp1 in the majority of cells. The assay was conducted in H1299, a lung-derived adenocarcinoma cell line previously used in COVID-19 research^7,15^. Using GFP as a marker, > 95% of H1299 cells are routinely transfected using the mRNA lipofection method.

**Figure 1:**
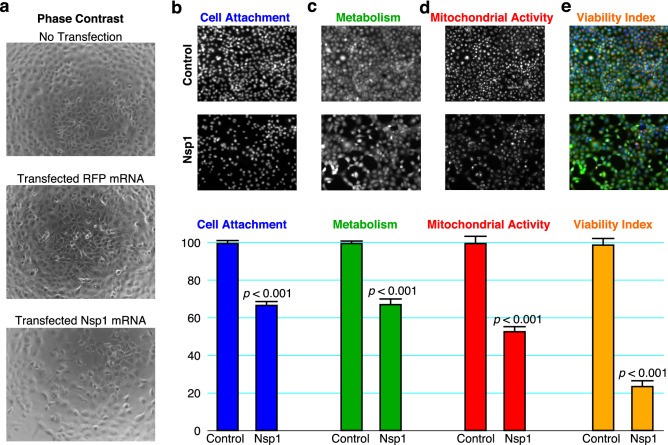
Quantitation of the cytopathic effects of Nsp1 in H1299 cells. H1299 cells were transfected with Nsp1 mRNA in 96-well plates as described in “Methods” section. (**a**) Phase contrast images of non-transfected cells, and cells transfected with Tag-Red Fluorescent Protein (RFP) mRNA as a control or Nsp1 mRNA. Both RFP and Nsp1 mRNAs were flanked by viral UTRs, and cells were transfected under identical conditions on the same plate. Control (non-transfected) and Nsp1-transfected cells were stained with (**b**) Hoescht 33,342 dye as a measure of cell attachment; (**c**) Calcein-AM, as a measure of vitality or metabolic diversion; (**d**) TMRE, as a measure of mitochondrial membrane potential or activity. Fluorescent images and quantitation (*n* = 6; *p* value from a two-sided t-test compared to the control) are depicted. (**e**) The Viability Index is the normalized product of quantitation using the latter three dyes. The images in (**e**) were pseudocolored blue (Hoescht), green (Calcein-AM) and red (TMRE).

Cell death is readily apparent in phase contrast images of H1299 cells 1 day after transfection with Nsp1 mRNA (Fig. 1a). 60–70% of the cells remain adhered to the surface of the plate (Fig. 1b), indicating that 30–40% of cells die within a day after Nsp1 expression. Determination of metabolic viability using the stain, calcein-AM^16^, which is a measure of intracellular esterase, revealed that overall metabolism declined to 60–70% with Nsp1 expression (Fig. 1c). Nsp1-transfected cells also displayed a significant decline in mitochondrial membrane integrity, an early marker for apoptosis, to about 50–60% (Fig. 1d). Thus, 3 independent measures of cell viability are negatively affected by the expression of Nsp1.

To develop a quantitative measure of Nsp1 action, we sought to consolidate the independent measurements of cell viability mentioned above. While each independent measurement (cell adherence, metabolism, and mitochondrial membrane integrity) was significantly reduced in Nsp1-transfected cells (Fig. 1b–d), the magnitude of the reduction in each case was too variable to permit confident identification of Nsp1 inhibitors using a single measurement. It is likely that cell death is gradual and proceeds through stages once the cell’s translational apparatus is subverted. Indeed, Nsp1 expression led to greater cell death when allowed to proceed for 48 or 72 h^7^; however, a longer time in cell culture would increase the assay time and introduce confounding variables (i.e. cell growth) into the procedure.

The 3 measurements of cell health are quantitated by fluorescent dyes with distinct excitation/emission spectra, permitting the simultaneous capture of multiplexed data (Fig. 1b–e). Images of transfected cultured cells reveal that the 3 independent measures of cell health are not uniform throughout the population (Fig. 1b–e). Thus, combining the quantities should reduce the variability. Accordingly, we define a measure that incorporates all three quantities, which we term the “Viability Index”. The Viability Index is the product of all three measurements, normalized to 100 for healthy, non-transfected cells. As shown in Fig. 1e, the Viability Index shows a robust difference between Nsp1-transfected cells and healthy non-transfected cells, with minimal quantitative variability.

**Figure 2:**
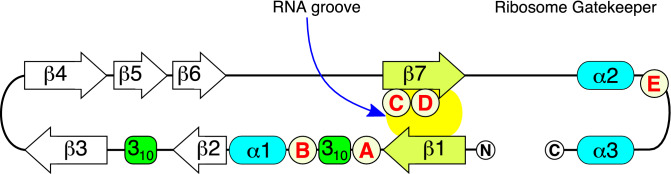
Secondary structure of Nsp1. Schematic diagram depicting the secondary structure of Nsp1 represented as an unfolded chain (from N- to C-terminal) with numbered alpha helices (α), numbered beta sheets (β), and 3_10_ helices. The beta sheets, β2 pairs with β6, β3 pairs with β4, and β1 pairs with β7 to form an RNA groove. The C-terminal helix-loop-helix (α2-α3) is termed the ribosome gatekeeper^13^ because of its role in blocking the RNA tunnel in the 40S ribosomal subunit. The sequential order and beta sheet pairings were adapted from Semper et al.^18^ and Almeida et al.^19^. The mutations depicted are (**A**) (L21M), (**B**) (D33R), (**C**) (L123S/R124E), (**D**) (N128S/K129E), and (**E**) (K164A).

**Figure 3:**
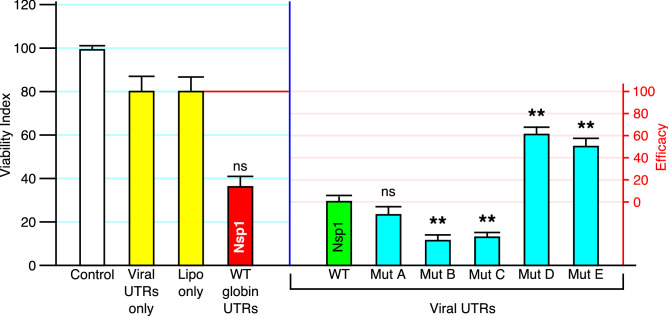
Measurement of efficacy and relationship to Nsp1 mutants. The Viability Index was determined for H1299 cells treated under various control conditions: no transfection (Control), transfected with RNA containing only viral 5′ and 3′UTR and no coding sequence (Viral UTRs only), or transfected with no RNA (Lipo only). Nsp1 mRNA was also transfected into H1299 cells in different contexts: wild-type Nsp1 flanked by the 5′UTR and 3′UTR corresponding to that of human alpha-globin mRNA (WT globin UTRs), or flanked by the viral UTRs corresponding to that of SARS-CoV-2 (WT Viral UTRs). All point mutations of Nsp1 (A through E, corresponding to the point mutations described in Fig. 2) were expressed as coding regions flanked by Viral UTRs. Efficacy is defined by a different scale as indicated, and represents the degree to which wild-type Nsp1 toxicity is reversed compared to H1299 cells incubated under the same conditions of transfection. The statistical significance of context and mutations compared to WT Nsp1-Viral UTRs are indicated (*n* > 15 for each measure; *p* value from a two-sided t-test, ns = not significant, **p < 10^−5^).

The 2D secondary structure (Fig. 2) and 3D crystal structure^17,18^ of Nsp1 from SARS-CoV-2 have been determined. Nsp1 is 180 amino acids long and folds into an N-terminal globular domain that contains 7 beta sheets (residues 1–120), connected by an unstructured linker to a helix-loop-helix region in the C-terminal domain (residues 121–180).

The globular N-domain contains an RNA groove that accommodates the 5′UTR of viral RNAs^9^. The RNA groove is created by juxtaposition of the first and last beta sheet of this region^9^ (Fig. 2), and mutations within the groove almost always diminish the action of Nsp1^20^, strongly suggesting that the RNA-binding groove in the N-domain is a functional site.

The C-terminal helix-loop-helix domain fits into the RNA tunnel of ribosomes, thereby blocking host cell translation^8^. Mutations affecting this domain also attenuate the activity of Nsp1^20,21^. This region has been termed the “ribosome gatekeeper” because it binds to ribosomes and promotes translation of viral mRNA^13^.

Thus, at least two functional sites are defined by mutational analysis within Nsp1: the RNA groove in the N-domain and the C-terminal helix-loop-helix. Both sites are potential targets for drug development.

To validate the utility of the Viability Index as a quantitative measure of Nsp1 activity, we measured the Viability Index of Nsp1 mRNA flanked by different untranslated regions, and Nsp1 mRNA containing different point mutations.

The context of the Nsp1 coding sequence impacts its toxicity. During infection, Nsp1 is transcribed from mRNA consisting of the coding region flanked by the 5′UTR (untranslated region) and 3′UTR of the SARS-CoV-2 genomic RNA^22^. These UTRs are present in all viral mRNAs^22^. Nsp1 mRNA flanked by viral UTRs had greater toxicity compared to Nsp1 in which the UTRs are replaced with those corresponding to the human alpha-globin gene (Fig. 3). This is consistent with the finding that Nsp1 binds to viral 5′UTRs and enhances the translation of the coding sequence^9^. Through another mechanism that is not well understood, Nsp1 also causes the degradation of host cell mRNAs through non-recognition of the 5′UTR^23,24^. This likely explains why Nsp1 flanked by globin UTRs is less toxic that Nsp1 flanked by viral UTRs.

Point mutations within the Nsp1 coding region had either no effect, *loss-of-function*, or *gain-of-function*. The relevant mutations are mapped to the 2D structure of Nsp1 (Fig. 2).

Mutation A (L21M) is an inadvertent mutation created outside of the RNA groove and had no apparent effect on Nsp1 activity as measured by the Viability Index (Fig. 3). Mutation B (D33R) is an established *gain-of-function* mutation that was previously shown to potently block host cell mRNA translation, consequently inhibiting interferon production by the SARS-CoV Nsp1^20^. The 3D model of Nsp1 indicates that D33 lies in close proximity to the RNA groove and may increase the binding affinity of the viral 5′UTR for this pocket. This mutation also led to a significant reduction of the Viability Index compared to wild-type Nsp1.

C (L123A/R124E) and D (N128S/K129E) are neighboring mutations located within the RNA groove (Fig. 2). D (N128S/K129E) is a well-characterized null mutation that blocks Nsp1’s ability to suppress interferon activity in several studies^7,8,20^. By contrast, the neighboring mutation had the opposite effect: a *gain-of-function* that increases Nsp1 toxicity. The results suggest that the RNA groove is a functional site, in which mutations can lead to potent and sometimes diametrically opposite effects on the action of Nsp1.

Mutation E (K164A) is located in the C-terminal domain (Fig. 2) and was previously reported to abolish the ability of Nsp1 to suppress host cell defenses^21^. The function of the C-domain is to block the RNA tunnel of ribosomes^8,11,13^. K164A also led to a significant reduction of the Viability Index compared to wild-type Nsp1 (Fig. 3). Thus, mutations define an essential role for this domain despite its small size (80 residues).

Further controls indicate that the conditions for introducing RNA into H1299 cells had effects on the Viability Index. Lipofectamine and UTR sequences both reduced the Viability Index. We therefore define Efficacy as the ability of a compound to reverse the effects of Nsp1 to the level observed in the absence of Nsp1 under the same conditions (Fig. 3). Thus, an Efficacy of 100% is the Viability Index when RNA from a construct containing only Viral UTRs is transfected, which is essentially an “empty vector” control. Similarly, an Efficacy of 0% is the Viability Index when RNA from an Nsp1 vector is transfected. Any compound exhibiting anti-Nsp1 activity should yield an Efficacy > 0. Using this as a scale, a compound simulating the well-characterized null mutations D (N128S/K129E) and E (K164A) would have Efficacies of 60% and 54% respectively.

The assay for Nsp1 activity was designed to simulate the early phase of COVID-19 infection in lung cells. Previous cell culture experiments utilize an MOI of 0–3, that is, up to 3 virions per adherent cell^25,26^. It is estimated that during infection, about 10^3^ infectious virions are eventually generated per cell^27^. By comparison, we calculate the number of Nsp1 mRNA molecules introduced into each cell is about 5 × 10^6^ copies, which is several orders of magnitude greater than the number of Nsp1 transcripts introduced per cell in live viral studies. This excessive Nsp1 expression accelerates H1299 cell death in this assay, but also ensures a stringent screen for potential Nsp1 inhibitors.

### Virtual screening for inhibitors of Nsp1

To experimentally evaluate a manageable number of candidate inhibitors using the Viability Index, candidates were stratified by virtual screening of compound libraries using previously determined 3-dimensional structures of Nsp1. The two functional sites within Nsp1 (described above) were the focus of this in silico screen: the RNA groove in the N-domain and the C-terminal alpha-loop-helix. Two crystal structures for the N-domain, 7k7p^17^ and 7k7n^18^, as well as three structures for the C-domain, 6zlw^8^, 6zok^11^, and 7k5i^12^, were used.

Two established algorithms were used to stratify compound libraries: Internal Coordinate Mechanics (ICM)^28^ and AutoDock Vina^29^. ICM stratifies compounds using parameters that assume both the ligand and protein receptor are flexible. AutoDock Vina determines the theoretical binding affinity of compounds but assumes the 3D structure of the protein receptor is fixed^29^. Data from both types of calculations differ, but results from both were used to guide subsequent experimental assays.

ICM was used to screen a database of approved drug molecules^30^, focusing on the RNA groove region represented in the crystal structure, 7k7p^17^. Due to the small size of the C-domain, it was not possible to use ICM to screen the database of approved drug molecules for potential inhibitors.

Autodock Vina was used to virtually screen publicly available compound databases: FDA-approved and World-approved drugs in the ZINC15 database^31^, eDrug-3D^32^, and selected compounds from PubChem^33^. Autodock Vina was applied to compound interactions with both the RNA groove in the N-domain and the helix-loop-helix region in the C-domain to stratify potential inhibitors.

Due to the urgent need to identify potential therapeutics rapidly, only compounds that are readily available with documented human data, and with a theoretical binding affinity exceeding a specified threshold, were used in subsequent Nsp1 assays (Table 1).

**Table 1:** Compounds selected by virtual screening for evaluation in Nsp1 assays.

Compound	RNA groove ICM score	Kcal/mol	Kcal/mol	Kcal/mol	
		Open grid N-domain	RNA groove N-domain	Open grid C-domain	
**Selected using ICM**					
Eravacycline	− 28.08	− 6.7	− 6.6	− 6.6	
Selumetinib	− 26.81	− 5.6	− 5.8	− 5.7	
Cyclo(L-His-L-Pro)	− 25.62	− 5.5	− 5.3	− 5.3	
Sulfasalazine	− 25.15	− 6.5	− 6.3	− 7.0	
Olsalazine	− 24.76	− 6.4	− 6.5	− 5.7	
Acelarin	− 24.39	− 6.2	− 6.7	− 6.1	
WP 1066	− 24.24	− 6.0	− 6.3	− 6.0	
Sulbactam	− 23.99	− 5.0	− 5.0	− 4.2	
Flufenoxuron	− 23.98	− 7.6	− 6.5	− 7.4	
Cabotegravir	− 22.82	− 6.7	− 7.5	− 6.7	
**High affinity for the N-domain**					
Zafirlukast	> − 15	− 7.9	− 8.3	− 8.3	
Eltrombopag	> − 15	− 7.7	− 8.1	− 7.7	
Imatanib	> − 15	− 7.4	− 8.0	− 7.5	
Beta-Carotene	> − 15	− 7.8	− 7.8	− 6.7	
Venetoclax	> − 15	− 7.6	− 7.7	− 8.1	
Ponatinib	> − 15	− 7.3	− 7.7	− 7.4	
Montelukast	> − 15	− 7.5	− 7.7	− 7.2	
Ergoloid Mesylate	> − 15	− 8.0	− 7.3	− 6.6	
Digotoxin	> − 15	− 7.6	− 7.2	− 7.6	
**High affinity for the C-domain predominantly**					
Pazopanib	> − 15	− 6.8	− 6.8	− 7.6	
Rilpivirine	> − 15	− 6.7	− 5.9	− 7.5	
Atovaquone	− 18.74	− 6.4	− 6.2	− 7.2	
Brigatinib	> − 15	− 6.4	− 6.8	− 7.2	
**High affinity for the Holo N-domain**					
Cepharanthine	> − 15	− 8.0	− 6.8	− 6.6	
Rapamycin	> − 15	− 8.0	− 5.2	− 6.4	
Milbemycin oxime	> − 15	− 7.9	− 6.4	− 7.2	

Compound	RNA groove ICM score	Kcal/mol	Kcal/mol	Kcal/mol	Kcal/mol
		Open grid N-domain	RNA groove N-domain	Open grid C-domain	Previously published
**Previously investigated**					
Tirilazad^9,34^	> − 15	− 8.5	− 7.9	− 8.0	− 10.4
Lumacaftor^34^	> − 15	− 8.2	− 8.4	− 7.7	− 9.6
Golvatinib^34^	> − 15	− 8.9	− 7.8	− 7.7	− 9.6
Glycyrrhizic acid^9,35^	> − 15	− 7.0	− 6.0	− 6.8	− 9.24
Dihydroergotamine^34^	> − 15	− 8.8	− 8.8	− 7.7	− 8.9
Nilotinib^34^	> − 15	− 7.7	− 7.4	− 8.4	− 8.8
Conivaptan^34^	> − 15	− 8.3	− 6.6	− 7.9	− 8.7
Radotinib^34^	> − 15	− 8.1	− 7.2	− 8	− 8.5
Rimegepant^34^	> − 15	− 7.6	− 7.6	− 6.4	− 8.4
**Other**					
Risperidone	− 15.89	− 7.2	− 7.1	− 6.9	
Haloperidol	> − 15	− 5.5	− 6.3	− 6.5	
Amphotericin B	> − 15	− 7.5	− 5.7	− 7.0	
Ampicillin	> − 15	− 5.9	− 6.3	− 5.4	
Tetracycline	> − 15	− 6.2	− 5.8	− 5.9	
Penicillin	> − 15	− 5.8	− 5.2	− 5.8	
Streptomycin	> − 15	− 5.5	− 5.8	− 5.1	
Kanamycin	> − 15	− 5.4	− 5.1	− 5.3	

About 12,000 compounds in a database of approved drug molecules were screened by ICM and stratified by ICM score. Readily available compounds with an ICM score < − 22 were selected for experimental testing. About 6500 compounds were screened by AutoDock Vina as described in “Methods” section. Readily available compounds with a binding score < − 7.2 were selected for experimental testing. Compounds identified from previously reported screens^9,34,35^ are listed with both published scores and scores derived from this study. Commonly used compounds listed in the “other” category have ICM or Vina scores in a range suggesting that they do not interact with Nsp1, and were used as negative controls.

### Individual compounds partially reverse Nsp1 toxicity

Compounds were serially diluted and applied to Nsp1-transfected H1299 cells, and the Viability Index and Efficacy calculated. In all cases, the maximum Efficacy of the drug (Emax) rarely exceeded 20% and multiple measurements were obtained over a range of concentrations. Due to the low value of Emax, the EC50 could not be reliably calculated, so the EC100 (concentration at which the maximum efficacy was observed) was used instead. The half-maximal cytotoxic concentration (CC50) was also determined using the Viability Index over a higher range of drug concentrations. The Safety Index was then calculated as the ratio of CC50/EC100. These data are shown in Table 2.

**Table 2:** Viability indices of selected compounds.

Compound	EC100 (µM)	Emax	%CV mean	*N*	CC50 (µM)	Safety index
**Selected using ICM**						
Eravacycline	0.51	7.5	67	4	3	6
Selumetinib	0.13	3.1	89	3	43	329
Cyclo(L-His-L-Pro)	32.0	6.4	67	6	> 80	> 3
Sulfasalazine	0.02	7.2	91	3	> 80	> 3800
Olsalazine	0.03	4.3	92	4	> 8	> 240
Acelarin	0.33	7.3	141	2	59	179
WP 1066	0.82	7.1	61	3	7	8
Sulbactam	0.13	4.9	57	4	> 80	> 600
Flufenoxuron (†)	0.48	3.4	141	2	> 80	> 160
Cabotegravir (†)	20.2	4.7	71	3	82	4
**High affinity for the N-domain**						
Zafirlukast		0.0		3	Toxic	Toxic
Eltrombopag	0.01	3.8	34	3	> 8	> 600
Imatanib (†)	2.82	10.8	44	7	24	8
Beta Carotene (†)	0.18	1.8	141	2	> 16	> 89
Venetoclax (†)	0.06	5.8	100	4	24	412
Ponatinib (†)	0.10	18.0	33	20	4.1	33
Montelukast (†)	0.63	13.1	29	19	8.1	13
Ergoloid Mesylate (†)	0.14	3.4	114	3	> 0.8	> 6
Digotoxin		0.0		1	Toxic	Toxic
**High affinity for the C-domain predominantly**						
Pazopanib (†)	1.15	5.4	70	6	> 160	> 139
Rilpivirine (†)	0.05	19.1	38	14	1.7	33
Atovaquone (†)	0.12	0.4	112	5	0.8	7
Brigatinib (†)	0.02	5.5	122	3	0.8	34

Compound	EC100 (µM)	Emax	%CV mean	*N*	CC50 (µM)	Safety index
**High affinity for the Holo N-domain**						
Cepharanthine (†)	2.86	1.1	122	3	> 8	> 3
Rapamycin (†)	2.5	0.3	115	4	16	6
Mibemycin oxime	0.01	6.3	83	5	> 8	> 600
**Previously investigated**						
Tirilazad	2.05	13.3	58	5	132	64
Lumacaftor (†)	2.30	5.0	55	4	270	118
Golvatinib (†)	0.15	11.4	66	4	20	136
Glycyrrhizic Acid (†)	1.11	13.4	122	3	> 160	> 145
Dihydroergotamine (†)	0.13	8.0	141	2	4.8	38
Nilotinib (†)	0.18	3.7	6	2	> 40	> 222
Conivaptan (†)	1.3	23.8	93	2	25	19
Radotinib	0.13	10.6	35	5	> 80	> 600
Rimegepant	0.13	8.3	99	4	> 80	> 600
**Other**						
Hygromycin	0.021	1.7		1	> 80	> 3800
Ampicillin	2.05	5.3	134	2	> 80	> 40
Tetracycline	80.0	4.9	110	2	> 80	> 1
Zeocin	0.33	7.8		2	> 80	> 240
Doxycycline	5.12	15.3		1	> 80	> 15

Compounds were serially diluted in DMEM-N2 and applied to Nsp1-transfected H1299 cells in 96-well plates as described in “Methods” section. The maximum Efficacy (Emax) and concentration at which Emax was observed (EC100) were determined over *N* replicate experiments as indicated. The %CVmean is the ratio of standard error of the mean (SEM) to the mean and expressed as a percentage. The half-maximal cytotoxic concentration (CC50) of each compound was also determined using the Viability Index. The Safety Index is defined as the ratio of CC50 over EC100. Data for compounds determined under serum-free conditions (i.e. DMEM-N2) are marked (†), while all other data were determined in cells incubated with fetal calf serum.

None of the compounds tested displayed a robust ability to reverse the toxic effects of Nsp1. Due to the generally low Efficacy shown by most compounds, high variability was observed in calculations of Efficacy as shown by %CVmean values. However, selected compounds may have synergistic inhibitory effects on Nsp1. Thus, compounds with a high Emax, low %CVmean, low EC100, and a high Safety Index were considered for further investigation (Table 2).

It is noteworthy that some of the candidates selected for this analysis were identified in previous in silico work, and have reported affinities that were much greater than those calculated here (Table 1). Previously investigated candidates^9,34,35^ were docked to a simulated 3D structure of SARS-CoV-2 Nsp1 before the experimentally derived 3D structure was available, which may explain why the reported theoretical binding affinities were high. Despite the appeal of high theoretical binding affinities to Nsp1, none of the most promising candidates identified by others or by our studies could reverse the toxic effects of Nsp1 in a meaningful fashion.

### Synergistic Nsp1-inhibitory interactions among compounds

Two potentially functional sites within Nsp1 were used to screen for potential inhibitors of Nsp1: the N-terminal RNA groove and the helix-loop-helix C-terminal region. Most of the compounds that are thought to bind to the N-terminal RNA groove also have high binding affinities for the C-terminal region (Table 1). However, there are compounds that possess high affinity for the C-terminal region with negligible affinity for the N-terminal RNA groove (Table 1). These compounds may bind to sites in the C-terminal region that differ from those that with highest affinity for the RNA groove. Accordingly, synergy may exist between compounds that bind to either site *preferentially*. In addition, the RNA groove can accommodate several compounds, raising the possibility of synergistic binding to this region.

Compounds from the original list (Table 1) were selected for synergistic studies (Table 2). Preferred compounds had an Emax with a low %CVmean, an EC100 in the low micromolar or sub-micromolar range, and a Safety Index > 5. Drug combinations containing serial dilutions of each compound were applied to Nsp1-transfected cells in a 2D matrix on 96-well plates. The efficacy of each combination was determined and data visualized using the online tool, SynergyFinder^36^. To quantify synergistic interactions, the ZIP scoring method was used^37^.

Due to the need to progressively acquire greater numbers of data points to determine synergy as the number of compounds increases, only the first significant synergistic interactions that are of clinical significance are reported at this time. We initially examined potential interactions among compounds that are thought to bind with high affinity to the N-terminal RNA groove. Cursory analyses of combinations that involved Olsalazine, Eravacycline, Dihydroergotamine, Montelukast, Ponatinib, Imatinib, Venetoclax, Nilotinib, and Golvatinib revealed weak or non-existent synergistic or additive interactions, though the analyses were not exhaustive. One compound that attracted our attention was Montelukast, which showed a broad tendency to enhance Efficacy in multiple cases (Supplementary Figs. 1, 2). Montelukast + Ponatinib was among the first drug combinations identified that consistently displayed higher Efficacy than either drug alone, although the effect was primarily additive (Supplementary Fig. 1a). Another compound that consistently raised Efficacy in a synergistic pattern was Tirilazad, which was reported to bind tightly to the RNA groove in previous screens^9,34^. Tirilazad combined with either Montelukast (Supplementary Fig. 1b) or Ponatinib (Supplementary Fig. 1c) substantially raised Efficacy, but the concentration of Tirilazad (1 to 5 µM), its limited oral bioavailability, and its status as an investigational drug are obstacles to its further clinical development.

The low Efficacies observed using pairs of compounds prompted us to consider adding a third drug to Montelukast + Ponatinib (fixed at a molar ratio of 10:1). Indeed, adding Conivaptan (Supplementary Fig. 2a) or Tirilazad (Supplementary Fig. 2b) substantially raised Efficacy. In the latter case, the highest Efficacies ranged from 45 to71%, suggesting it is possible to find a drug combination that reverses Nsp1 toxicity to the same extent as a null mutation in the gene itself.

We next asked if synergy exists between compounds *predominantly* targeting the C-terminal domain and compounds targeting the N-terminal RNA groove (Tables 1,2), many of which also target the C-domain. We first tested combinations with Pazopanib, which had the highest theoretical affinity for the helix-loop-helix region (Table 1). Significant synergy was observed between Pazopanib and the Montelukast + Ponatinib combination (fixed at a molar ratio of 10:1) (Supplementary Fig. 2c). However, the concentration of Pazopanib required to attain Efficacies mimicking the effect of a null mutation was about 80 µM, precluding clinical use (Supplementary Fig. 2c).

**Figure 4:**
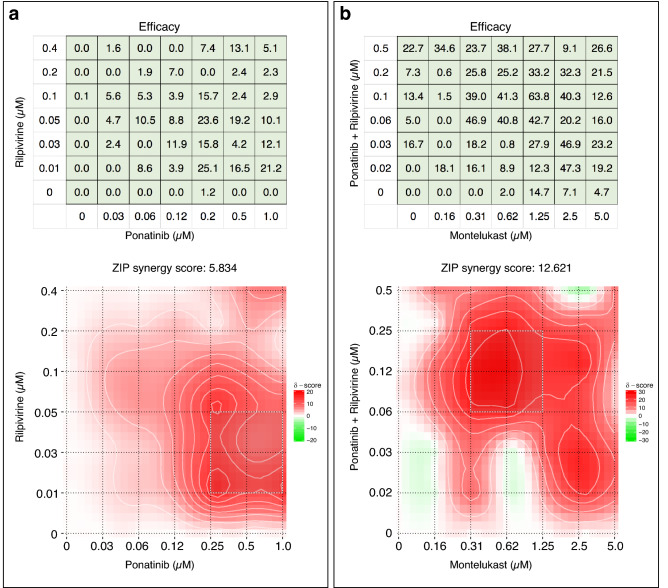
Synergistic interactions among Ponatinib, Rilpivirine, and Montelukast. Combinations of drugs were applied to Nsp1-transfected H1299 cells, and Efficacies was determined. The Efficacy at the indicated concentrations of each drug were used to visualize synergy using an online tool^36^. Areas demarcated in red represent synergistic combinations as shown in the scale quantitated by the ZIP method^36,37^. (**a**) Data were pooled from triplicate experiments to generate the Efficacy table and Synergy plot of Ponatinib and Rilpivirine. (**b**) Efficacy table and Synergy plot between a combination of Ponatinib + Rilpivirine at a molar ratio of 2.5 to 1, and Montelukast. The concentration for the Ponatinib + Rilpivirine combination reflects that of Ponatinib only.

We next tested combinations with Rilpivirine, which had the next highest theoretical binding affinity for the C-terminal helix-loop-helix region (Table 1). Significant synergy was observed between Ponatinib and Rilpivirine, well within concentrations that would be used clinically and over a broad concentration of each compound (Fig. 4a). Since the addition of Montelukast improved Efficacy in several experiments involving drug combinations (Supplementary Figs. 1a,b, 2), this drug was added to the Ponatinib + Rilpivirine combination (fixed at a molar ratio of 2.5:1). Substantial synergy was observed over a broad concentration of each drug (Fig. 4b), with further enhancement of Efficacy. Under optimal conditions, the Efficacy of the Montelukast + Ponatinib + Rilpivirine combination ranged from 47 to 64% (Fig. 4b), which is similar to the effects of a null mutation in the Nsp1 gene (Fig. 3). These data suggest that a Montelukast + Ponatinib + Rilpivirine drug combination reverses the toxic effects of Nsp1 at concentrations that are attainable clinically.

**Figure 5:**
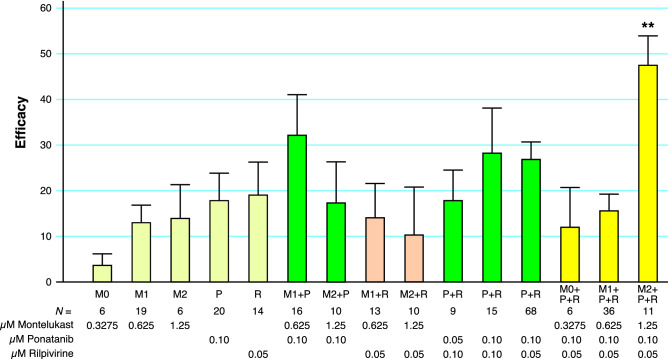
Comparative efficacies of drug combinations using Montelukast, Ponatinib, Rilpivirine. Efficacies were determined using compounds alone or in various combinations in Nsp1-transfected H1299 cells at the indicated concentrations. Measurements were conducted with *N* replicates and error bars represent ± SEM. One combination (M2 + P + R) displayed greater Efficacy than other combinations shown in this graph. A one-way ANOVA analyses indicates that there is significant difference between the means of at least two drug combinations (*F*_14, 243_ = 1.985, *p* = 0.02). Eliminating M2 + P + R from the analyses rendered the one-way ANOVA not significant (*F*_13, 233_ = 1.274, *p* = 0.23).

The Efficacies of Montelukast, Ponatinib, and Rilpivirine were examined systematically in Nsp1-transfected H1299 cells over several replicate experiments using concentrations that are attainable clinically (Fig. 5). Under these conditions, individual compounds displayed Efficacies < 20%, and various pairs showed marginal improvement. A one-way ANOVA was used to compare the Efficacies of the 15 drug combinations depicted in Fig. 5. The analyses revealed a statistically significant difference between the means of at least two drug combinations (*F*_14, 243_ = 1.985, *p* = 0.02). It is obvious that only the last drug combination (M2 + P + R) displayed substantial Efficacy compared to the others (Fig. 5). Eliminating M2 + P + R from the analyses rendered the one-way ANOVA not significant (*F*_13, 233_ = 1.274, *p* = 0.23). These analyses suggest that single drugs or pairs of drugs are unlikely to possess meaningful Efficacy. Moreover, among the combinations that utilized three drugs, only one combination showed effect.

**Figure 6:**
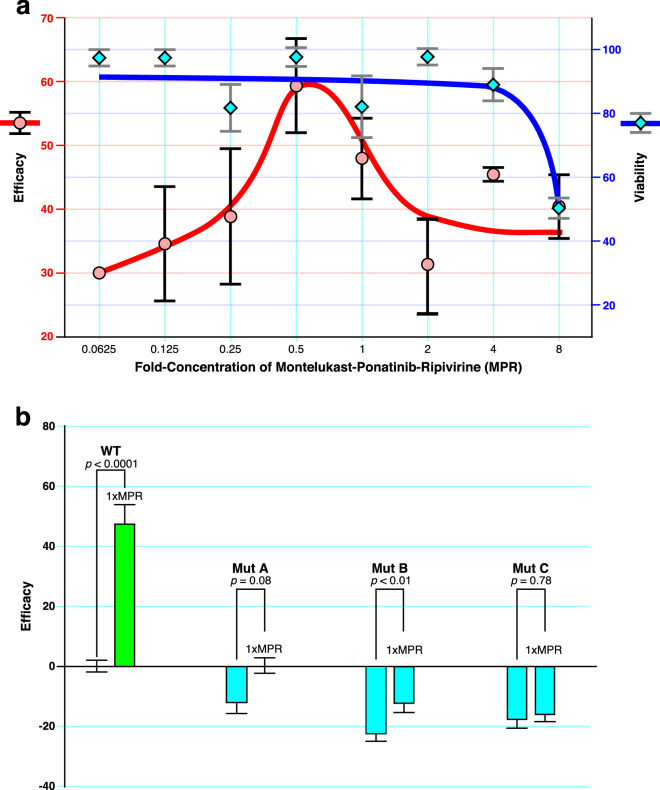
Dose–response of a Montelukast–Ponatinib–Rilpivirine (MPR) Combination. The ratio of Montelukast, Ponatinib, and Rilpivirine (MPR) were fixed such that a 1X concentration = 1.25 µM Montelukast + 0.1 µM Ponatinib + 0.05 µM Rilpivirine. (**a**) Varying concentrations of MPR were added to Nsp1-transfected H1299 cells and Efficacy determined and depicted. Cytotoxicity using the Viability Index in non-transfected H1299 were determined at various concentrations of MPR and depicted on the same graph. (**b**) 1 × MPR was applied to H1299 cells transfected with wild-type (WT) Nsp1 or the indicated Nsp1 mutations (Mut A–C). Error bars represent ± SEM. Statistical significance of treatment with 1xMPR is indicated (*n* > 5; *p* value from a two-sided t-test).

The data from Fig. 5 suggests that an optimal proportion of Montelukast + Ponatinib + Rilpivirine (abbreviated MPR) is 1.25: 0.10: 0.05. This triple combination was applied to Nsp-1 transfected H1299 cells over a range of concentrations (Fig. 6a). A typical dose–response curve is produced with serial dilutions of MPR, and the effective concentrations are well separated from toxic concentrations (Fig. 6a). The optimal concentration of MPR is actually 0.5x (0.625 µM Montelukast, 0.05 µM Ponatinib, and 0.025 µM Rilpivirine), resulting in a mean Efficacy of 59%. The CC50 of MPR was 8X, providing a safety index of 16 (Fig. 6a).

**Figure 7:**
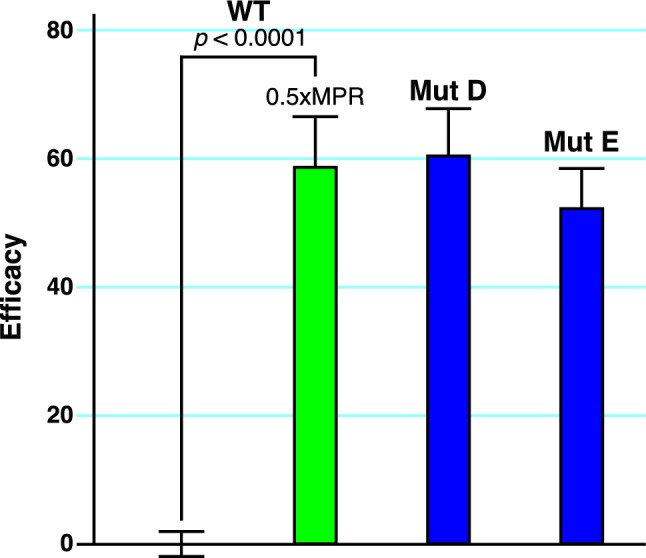
Comparison of the Anti-Nsp1 activity of MPR to null mutations. The efficacy of 0.5 × MPR on WT-Nsp1-transfected H1299 cells (*n* = 16, *p* value from a two-sided t-test) is compared to the effect of established null mutations in the Nsp1 gene (Mut D and E). The concentrations of Montelukast, Ponatinib, and Rilpivirine (MPR) at the 0.5X dilution are 0.625 µM Montelukast, 0.05 µM Ponatinib, and 0.025 µM Rilpivirine. There is no significant difference in the Efficacy of 0.5xMPR-treated WT-Nsp1-transfected H1299 cells compared to the effect of null mutations in Nsp1.

To further understand the mechanism by which the MPR drug combination may be acting, 1xMPR was applied to H1299 cells transfected with the Nsp1 point mutations, Mut A, B and C (Fig. 6b). In all cases, MPR treatment of H1299 cells transfected with these Nsp1 mutations failed to rescue these cells from toxicity. However, it is notable that 1xMPR raised Efficacy in the *gain-of-function* point mutant B (D33R), which lies outside of the RNA groove. By contrast, 1xMPRdid not raise Efficacy to a statistically significant level with mutations A and C, which are located adjacent to or within the RNA groove. These data suggest that the potential mechanism of MPR is to bind to the RNA groove of Nsp1. Lastly, the Efficacy of 0.5xMPR in treating WT-Nsp1-transfected H1299 cells is similar to the effect of the established null mutations, Mut D and E, in Nsp1 (Fig. 7). Given the importance of Nsp1 in the early pathogenesis of COVID-19, these data support the investigation of this repurposed drug combination in clinical trials for the treatment of this disease.

## Discussion

Nsp1 is a promising molecular target because of its critical role during early SARS-CoV-2 pathogenesis. In SARS-CoV-2^8,11,13^, SARS-CoV^38^, and MERS^39^, Nsp1 shuts down host protein synthesis but ribosomes remain permissive for viral protein synthesis. Experimental deletion of Nsp1 in a highly virulent beta-coronavirus, murine hepatitis virus, converts the virus from a lethal pathogen to a nonlethal one^40^. Moreover, naturally occurring variants of SARS-CoV-2 containing deletions in the helix-loop-helix region of the C-domain of Nsp1 have been identified in China^41^. This variant renders the virus less severe clinically, with lower viral loads and smaller plaque size^41^. These observations suggest that targeting Nsp1 of SARS-CoV-2 could mitigate the severe clinical sequelae of COVID-19 infection.

The mechanism by which SARS-CoV-2 induces apoptosis in lung cells has not been fully elucidated, but targeting this process can attenuate disease severity^42^. Among the most prominent host protein changes in SARS-CoV-2 infected alveolar epithelial cells are those affecting eukaryotic translation elongation and viral mRNA translation^43^, consistent with Nsp1’s primary action of subverting host protein synthesis^8,11,13^. Accordingly, Nsp1 blocks the production of interferon I^44–46^ and interferon III^23^, key players in the innate defense against viral infection. Mutations affecting the RNA groove or the helix-loop-helix region of Nsp1 reverse the interferon-blocking actions of Nsp1^20^. Thus, Nsp1 contributes to the apoptotic process by inhibiting host protein translation and interferon action. The Nsp1 assay described here is essentially a cytopathic assay that simulates the expression of Nsp1 mRNA during infection.

Since Nsp1 functions only when introduced inside cells, its actions are not dependent on viral tropism. Indeed, we observed similar actions of Nsp1 on HeLa cells (data not shown), a cell line that does not support SARS-CoV-2 replication^47^. SARS-CoV-2 infection can lead to multi-organ damage, and Nsp1 is a likely contender in this pathogenesis. The design of the assay described herein can be adapted to investigate the role of Nsp1 in other tissues.

Virtual screening of compound libraries is useful for ranking the likelihood that a chemical interacts with a specified target, saving time and expense in drug screening. An estimated 5–12% of compounds from a “hit” list eventually turn out to have activity^48,49^. However, Nsp1 is not homologous to known mammalian proteins^50^, and algorithms may not be optimized for identifying potential “hits”. To increase our chances of finding “hits”, we used two different, well-regarded algorithms to compile an initial list of prospective Nsp1 inhibitors, focusing on readily available compounds that could be repurposed. In addition, compounds identified by other investigators^9,34,35^ were examined. However, experimentally, none of the compounds alone were capable of substantially inhibiting Nsp1 (i.e. Efficacy > 20%).

We reasoned that synergistic interactions among compounds could promote their ability to inhibit Nsp1. For instance, compounds that preferentially target different functional domains – such as the N-terminal RNA groove and C-terminal helix-loop-helix – may work synergistically together. However, identifying the relevant synergistic interactions is labor-intensive because the number of potential drug pairs multiplies as more drugs are tested, and synergy can only be determined after testing serial dilutions of each drug together in a 2 × 2 matrix.

Here, we report significant synergy with Montelukast, Ponatinib, and Rilpivirine, which together, abolishes Nsp1 toxicity in our cell-based assay. The combination is clinically meaningful for the following reasons. All three drugs are FDA-approved and can be administered orally once a day. For synergistic inhibition, the effective concentration ranges of Montelukast, Ponatinib, and Rilpivirine are 625–1,250 nM, 50–125 nM, and 30–60 nM, respectively. After a standard dose of 10 mg of Montelukast, peak plasma concentrations ranged from 495 ng/mL (810 nM) to 603 ng/mL (990 nM)^51^. The steady state plasma concentration of Ponatinib in patients taking the standard oral dose of 15 mg was 43.6 ng/mL or 80 nM^52^. The standard oral dose of Rilpivirine is 25 mg daily, resulting in plasma levels of 30–70 nM^53^. There are no known adverse interactions among the 3 drugs, though the doses may require reduction due to common paths of elimination according to information on DrugBank^30^. Thus, all three drugs can be given orally and are expected to attain plasma concentrations that in our preclinical study, inhibits Nsp1 to the same extent as a null mutation.

In addition, Montelukast, Ponatinib, and Rilpivirine have been suggested as treatments for COVID-19 by other studies. In silico docking studies suggest that Montelukast binds to the SARS-CoV-2 proteins Mpro^54^, RdRp^54^, 3CL^55^, as well as the C-terminal domain of Nsp1^56^. Afsar et al.^56^ reports that Montelukast binds to C-terminal domain of Nsp1 with an affinity of 10.8 µM and exerts anti-viral effects in cell culture at the same concentration. Another study in Vero-E6 cells showed that Montelukast inhibited SARS-CoV-2 replication at an IC50 of 18.82 µM^57^. However, the effective anti-viral dose of Montelukast needed in these studies is close to the lethal dose of the drug (LD_80_ = 20 µM) in Vero-E6 cells^56^. In addition, Montelukast inhibits the action of inflammatory cytokines, suggesting it could tame cytokine storms during severe COVID-19 infection^58^. No prospective trials of Montelukast have been performed, but a retrospective study suggests that COVID-19-positive patients taking this drug (10 mg daily) had fewer deleterious symptoms compared to patients not taking the drug^59^. Recent in silico docking studies also suggest that Ponatinib binds to host factors that influence infection^60,61^, and that Rilpivirine can bind to Mpro, PLpro, Spro, ACE2, and RdRp^62^.

This initial study suggests that detection of a single repurposed compound to inhibit Nsp1 to a clinically meaningful degree may be challenging. When the search strategy was expanded to include 2–3 drugs co-administered together, a number of potential combinations were identified. Specifically, a mixture of Montelukast, Ponatinib, and Rilpivirine was shown to inhibit Nsp1 toxicity in cultured lung cells at concentrations that are clinically attainable with oral administration. Further studies are needed to determine the utility of this finding by prospective clinical trials, as well as to identify other meaningful drug combinations.

## Methods

### Cell lines, cell culture, and transfection

Unless otherwise specified, all reagents were purchased from ThermoFisher Scientific. H1299 cells were obtained from the American Type Culture Collection (ATCC) and maintained in DMEM supplemented with 10% fetal calf serum (FCS) and 100 U/mL penicillin–streptomycin. Cells were grown in an incubator that maintained the temperature at 37 °C, air humidity at 95%, and CO_2_ concentration at 5%, and passaged every 3–4 days with PBS and 0.05% Trypsin–EDTA. To prepare cells for transfection, they were plated at a density that would be predicted to reach 50% the next day in a volume of 70 µL per well on 96-well plates.

RNA transfections were carried out in 96-well plates with H1299 cells plated at 50% density. The equivalent of 0.05–0.2 µL of Lipofectamine™ MessengerMax™ was first diluted in Opti-MEM™ in a volume of 5 µL for 5 min, and then added to 50–200 ng RNA (diluted in Opti-MEM to a volume of 5 µL) in a total volume of 10 µL, and incubated for an additional 10 min at room temperature. The mixture was then diluted to a volume of 50 µL with Opti-MEM and added to H1299 cells growing in a single well. This would be scaled depending on the number of wells to be transfected per plate.

### Nsp1 assay

H1299 cells growing on 96-well plates were transfected with Nsp1 mRNA as described above and incubated with the lipofectamine-RNA mix for 3 h. Little difference in gene expression was observed between 2 and 4 h of incubation with the lipofectamine-RNA mix. Media was then replaced by addition of 80 µL DMEM-10% FCS-100U/mL Pen-Strep or 80 µL serum-free DMEM-1% N2 supplement-100U/mL Pen-Strep. H1299 cells were then returned in the CO_2_ incubator overnight.

Approximately 20 h after replacement of the lipofectamine-RNA mix from H1299 cells, the media was again replaced with 100 µL of a mixture containing compatible fluorophores diluted in FluoroBrite™: 1 µM Hoechst 33,342, 1 µM Calcein-AM (Cayman Chemical Company), and 20 nM Tetramethylrhodamine, ethyl ester (TMRE, Cayman Chemical Company). Cells were placed in the CO_2_ incubator for 1 h and the media was changed to 50 µL of Fluorobrite or PBS alone.

Fluorescence from 96-well plates was measured using a Spectramax Microplate Gemini XPS reader with the following parameters: Hoechst 33,342 staining—excitation-355 nm; emission-460 nm; calcein-AM—excitation-485 nm; emission-520 nm; TMRE—excitation-544 nm; emission-590 nm. After normalizing values for each fluorescence reading to non-transfected controls, the product of all three readings represents the “Viability Index”.

### Efficacy, EC100, CC50

Efficacy is quantified as the degree to which a drug or drug combination reverses all toxic effects of Nsp1 as determined by the Viability Index. The quantity is a value between 0 and 100, where 0 represents no effect and 100 is complete reversal. The EC100 is the concentration of drug where maximum Efficacy is observed. The CC50 is the half-maximal concentration of drug that produces death in H1299 cells. The half-maximal concentration was determined from dose response data fitted to a sigmoidal curve (www.aatbio.com/tools).

### Statistics

Data were analyzed using the unpaired two-tailed t-test or analysis of variance (ANOVA). *p* < 0.05 was taken to be significant. Data were analyzed using Excel software.

### Ligand docking and screening

The ICM Pocket Finder method^63^ in ICM-Pro v3.9-1c (Molsoft, LLC) was used to define a druggable pocket within the Nsp1 crystal structure, 7k7p^17^. The pocket is essentially identical to the RNA groove that accommodates the 5′UTR of viral mRNAs^9^. The ICM-VLS method^64,65^ (MolSoft LLC) was used to dock, score and rank chemicals from the DrugBank database^30^ that are predicted bind to this pocket.

PyRx^66^ is a user interface that assimilates Autodock Vina^29^ with other programs, and was used to screen the compound libraries ZINC15^31^ and eDrug-3D^32^. The molecular targets were the RNA groove pocket within the structures, 7k7p^17^ and 7k7n^18^, and the loop-helix-loop regions of the C-domain (structures 6zlw^8^, 6zok^11^, and 7k5i^12^).

### Compounds

All compounds were obtained from the Cayman Chemical Company with some exceptions. Eravacycline was obtained from MedChemExpress. Flufenoxuron, kanamycin, tetracycline, ampicillin, haloperidol, and risperidone were purchased from the Sigma-Aldrich.

All compounds were dissolved in the recommended solvent (DMSO, DMF, alcohol, or water). Compounds were diluted in serum-free DMEM-N2 to the desired concentrations before addition to media.

### Identification of synergistic interactions

H1299 cells were plates on 96-well plates and transfected with Nsp1 mRNA as described above. Lipofectamine-RNA mixtures were replaced with 80 µL serum-free DMEM-1% N2 supplement-100U/mL Pen-Strep. 20 µL of serial dilutions of each drug (diluted in DMEM-1% N2 supplement-100U/mL Pen-Strep) were added in a matrix configuration, and the cells were incubated for another 20 h in the tissue culture incubator. Plates were then subjected to the multiplexed fluorescent assay described above, and the Variability Index and Efficacy over a range of drug concentrations were determined.

Efficacy measurements from a matrix of drug concentrations were evaluated using Synergy Finder 2.0^36^, an online visualization tool to identify synergistic interactions. The ZIP scoring method was used to calculate synergies^37^.

## Supplementary Information

Supplementary Information.

## Data Availability

All data generated or analyzed during this study are included in this published article (and its supplementary information files).
