# Supplementary material for: Synergistic interactions of repurposed drugs that inhibit Nsp1, a major virulence factor for COVID-19
Source: Sci Rep. 2022 Jun 17;12:10174. doi: 10.1038/s41598-022-14194-x (PMC9204075; doi:10.1038/s41598-022-14194-x)
Supplement: Supplementary file 1 — Supplementary Information. [file 41598_2022_14194_MOESM1_ESM.pdf]

Supplementary Figures

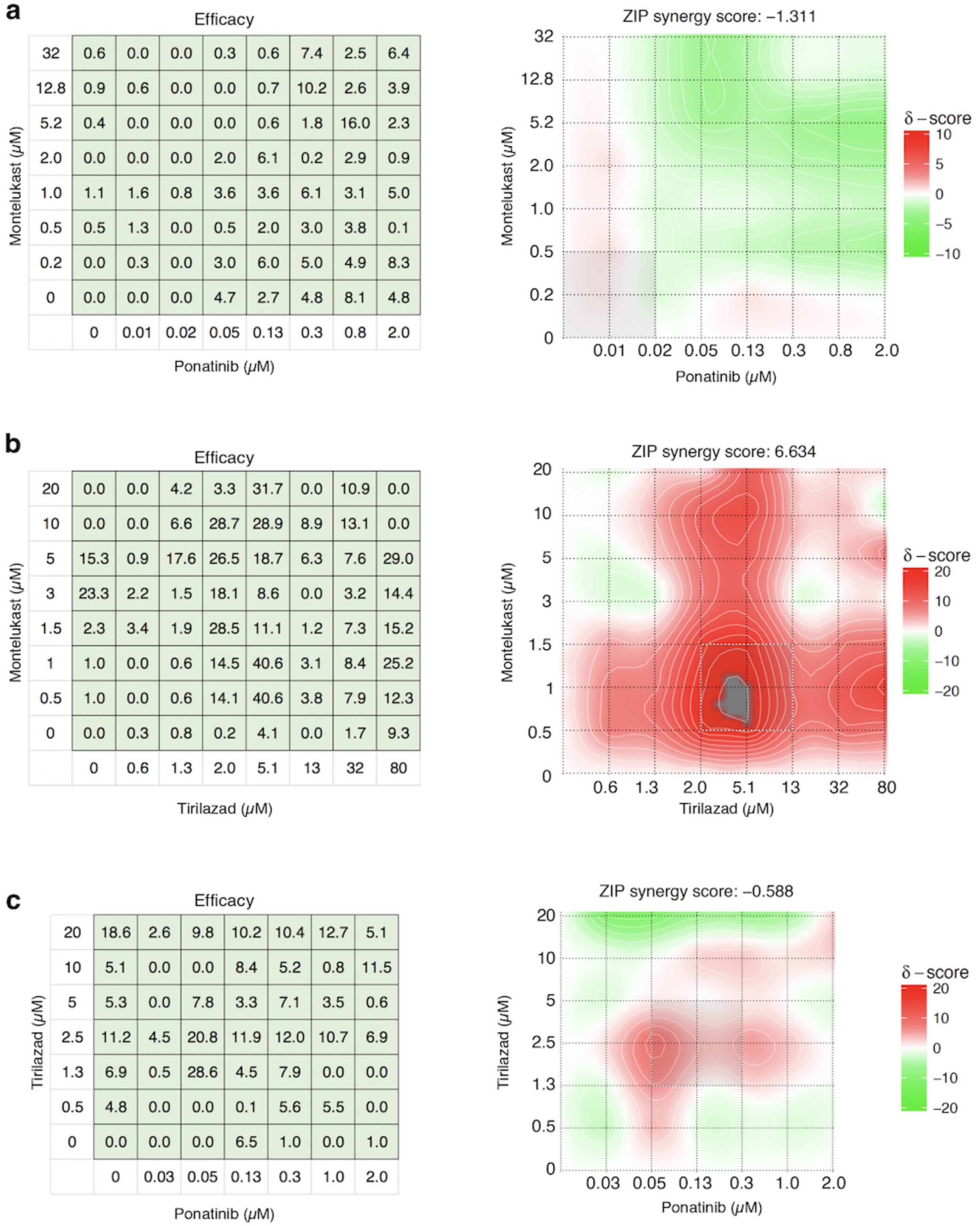

a Efficacy (rows: Montelukast (μM); columns: Ponatinib (μM))

32	0.6	0.0	0.0	0.3	0.6	7.4	2.5	6.4
12.8	0.9	0.6	0.0	0.0	0.7	10.2	2.6	3.9
5.2	0.4	0.0	0.0	0.0	0.6	1.8	16.0	2.3
2.0	0.0	0.0	0.0	2.0	6.1	0.2	2.9	0.9
1.0	1.1	1.6	0.8	3.6	3.6	6.1	3.1	5.0
0.5	0.5	1.3	0.0	0.5	2.0	3.0	3.8	0.1
0.2	0.0	0.3	0.0	3.0	6.0	5.0	4.9	8.3
0	0.0	0.0	0.0	4.7	2.7	4.8	8.1	4.8
	0	0.01	0.02	0.05	0.13	0.3	0.8	2.0

b Efficacy (rows: Montelukast (μM); columns: Tirilazad (μM))

20	0.0	0.0	4.2	3.3	31.7	0.0	10.9	0.0
10	0.0	0.0	6.6	28.7	28.9	8.9	13.1	0.0
5	15.3	0.9	17.6	26.5	18.7	6.3	7.6	29.0
3	23.3	2.2	1.5	18.1	8.6	0.0	3.2	14.4
1.5	2.3	3.4	1.9	28.5	11.1	1.2	7.3	15.2
1	1.0	0.0	0.6	14.5	40.6	3.1	8.4	25.2
0.5	1.0	0.0	0.6	14.1	40.6	3.8	7.9	12.3
0	0.0	0.3	0.8	0.2	4.1	0.0	1.7	9.3
	0	0.6	1.3	2.0	5.1	13	32	80

c Efficacy (rows: Tirilazad (μM); columns: Ponatinib (μM))

20	18.6	2.6	9.8	10.2	10.4	12.7	5.1
10	5.1	0.0	0.0	8.4	5.2	0.8	11.5
5	5.3	0.0	7.8	3.3	7.1	3.5	0.6
2.5	11.2	4.5	20.8	11.9	12.0	10.7	6.9
1.3	6.9	0.5	28.6	4.5	7.9	0.0	0.0
0.5	4.8	0.0	0.0	0.1	5.6	5.5	0.0
0	0.0	0.0	0.0	6.5	1.0	0.0	1.0
	0	0.03	0.05	0.13	0.3	1.0	2.0

Supplementary Figure 1. Synergistic Interactions of Potentially Promising Drug Pairs.

Serial dilutions of the indicated drugs were applied to Nsp1-transfected H1299 cells in 96-well plates, in a 2x2 matrix, and Efficacies was determined. The Efficacy at the indicated concentrations of each drug were inputted into SynergyFinder 2.0, an online tool for visualizing synergy [35]. Synergy is quantitated by the ZIP method [35, 36] and visualized on a red-green scale as indicated. Efficacy tables and Synergy plots are shown for each of the following drug pairs: (**a**) Montelukast versus Ponatinib (data were averaged or consolidated from 10 replicates); (**b**) Montelukast versus Tirilazad (data were averaged or consolidated from 3 replicates); and **(c)** Tirilazad versus Ponatinib (data were averaged or consolidated from 3 replicates).

a

Efficacy

Conivaptan (μM)						
83	12.9	20.9	27.5	20.4	0.0	0.0
33	0.0	1.9	9.6	6.3	0.0	0.0
13	0.0	7.8	1.9	0.0	0.0	0.0
5	0.0	0.0	0.0	0.6	0.0	0.0
2	0.0	6.7	0.0	0.0	0.0	0.0
0	0.0	1.5	1.6	0.0	0.0	0.0
	0	1	2	5	13	33

Montelukast-Ponatinib (μM)

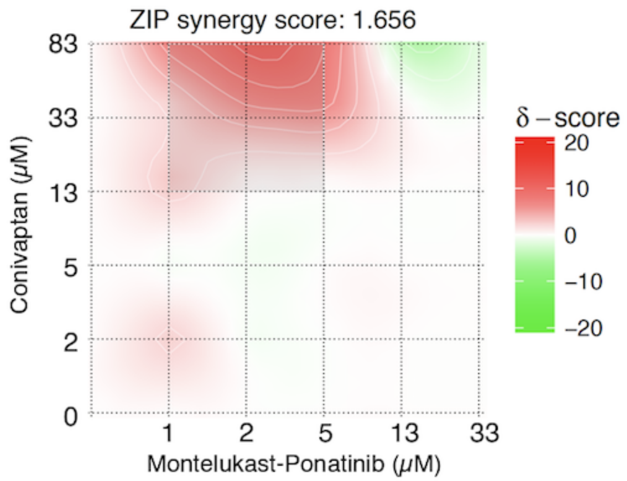

b

Efficacy

Tirilazad (μM)						
80	48.6	87.9	31.0	61.0	1.1	0.0
32	0.0	25.5	2.9	9.5	0.0	0.0
13	0.0	3.0	4.3	0.0	0.0	0.0
5	0.0	0.3	3.2	0.0	0.0	0.0
2	0.0	0.8	7.6	0.0	0.0	0.0
0	0.0	0.0	12.1	0.0	0.0	0.0
	0	1	2	5	13	33

Montelukast-Ponatinib (μM)

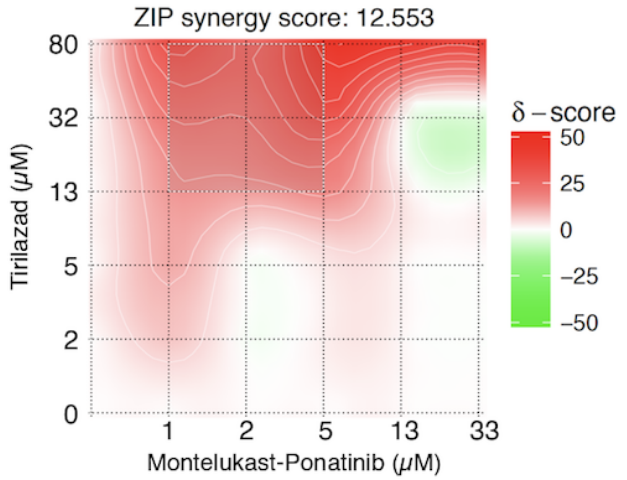

c

Efficacy

Pazopanib (μM)						
80	15.3	56.9	28.5	71.3	69.5	15.8
32	4.4	30.1	24.0	45.4	0.0	0.0
13	0.0	20.0	17.4	23.8	0.0	0.0
5	0.0	18.2	0.7	0.8	0.0	0.0
2	0.0	11.5	0.0	0.0	0.0	0.0
0	0.2	4.9	4.2	0.0	0.0	0.0
	0	1	2	5	13	33

Montelukast-Ponatinib (μM)

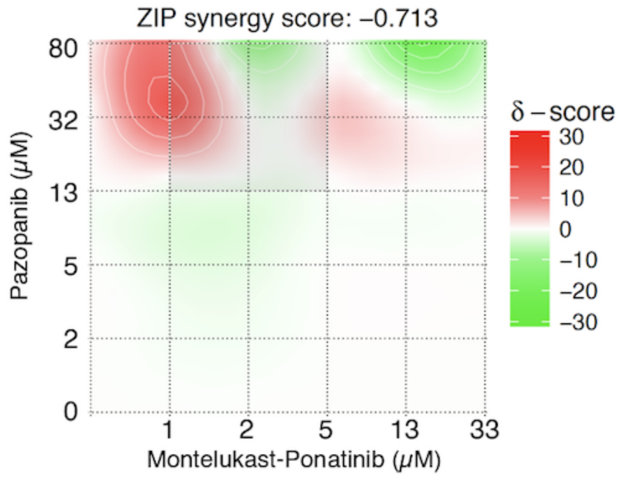

Supplementary Figure 2. Synergistic Interactions between Selected Drugs and Montelulast+Ponatinib (fixed molar ratio).

Serial dilutions of the indicated drugs and Montelukast+Ponatinib or MP (molar ratios fixed at 10:1) were applied to Nsp1-transfected H1299 cells in 96-well plates, in a 2x2 matrix, and Efficacies was determined. The concentration indicated for MP reflects that of Montelukast. Efficacy at the indicated concentrations of each drug were inputted into SynergyFinder 2.0, and synergy was quantitated and visualized as in Supplementary Fig. 1. Efficacy tables and Synergy plots are shown for each of the following drug combinations: (**a**) Conivaptan versus MP; (**b**) Tirilazad versus MP; (**c**) Pazopanib versus MP.
